# 1-(1-Benzyl-1*H*-benzimidazol-2-yl)ethanone

**DOI:** 10.1107/S1600536812043875

**Published:** 2012-11-14

**Authors:** Chuan-Jing Zhang, Xiu-Zhen Xu, Ning Yang, Ren-Ying Zhao, Yan-Qing Ge

**Affiliations:** a Taishan Medical University, Tai an 271016, People’s Republic of China

## Abstract

In the title compound, C_16_H_14_N_2_O, the benzimidazole ring system is essentially planar. The planes of the benzene rings make a dihedral angle of 85.92 (8)°. In the crystal, neighbouring molecule are connected into paris along the *c* axis by weak C—H⋯O interactions and the connected pairs are expanded through C—H⋯N hydrogen bonds and C—H⋯π interactions along the *b* axis.

## Related literature

For the synthesis, see: Cao *et al.* (2012 ▶). For applications of nitro­gen-containing heterocyclic compounds in the agrochemical and pharmaceutical fields, see: Ge *et al.* (2009 ▶, 2011 ▶). For a related structure, see: Sun *et al.* (2012 ▶).
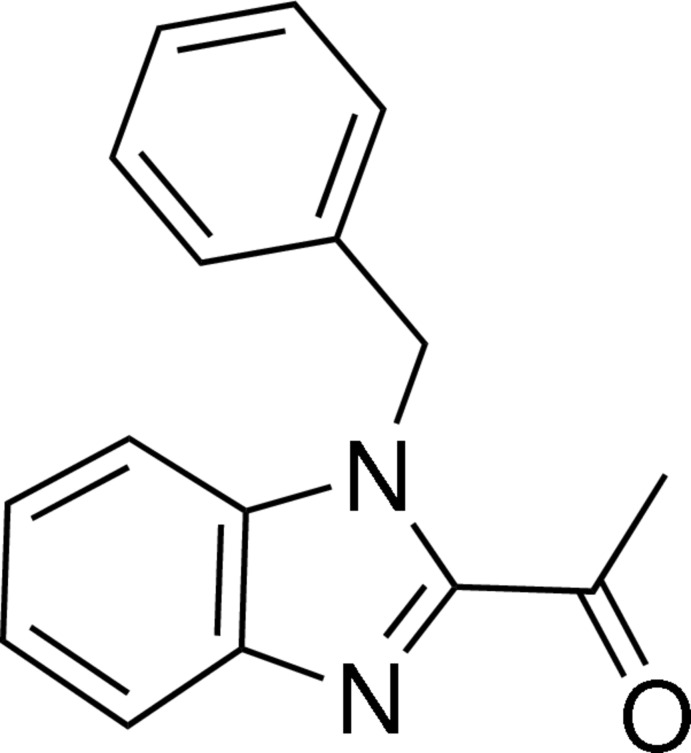


## Experimental

### 

#### Crystal data

C_16_H_14_N_2_O
*M* *_r_* = 250.29
Triclinic, 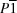

*a* = 6.1307 (10) Å
*b* = 6.5226 (12) Å
*c* = 34.739 (6) Å
α = 90.021 (3)°
β = 92.749 (3)°
γ = 110.674 (3)°
*V* = 1298.0 (4) Å^3^
*Z* = 4
Mo *K*α radiation
μ = 0.08 mm^−1^
*T* = 293 K
0.28 × 0.24 × 0.19 mm

#### Data collection

Brucker APEXII CCD area-detector diffractometer
Absorption correction: multi-scan (*SADABS*; Bruker, 2005 ▶) *T*_min_ = 0.978, *T*_max_ = 0.985
6666 measured reflections
4523 independent reflections
3775 reflections with *I* > 2σ(*I*)
*R*_int_ = 0.061

#### Refinement

*R*[*F* ^2^ > 2σ(*F* ^2^)] = 0.068
*wR*(*F* ^2^) = 0.189
*S* = 1.05
4523 reflections
345 parameters
H-atom parameters constrained
Δρ_max_ = 0.23 e Å^−3^
Δρ_min_ = −0.29 e Å^−3^

### 

Data collection: *APEX2* (Bruker, 2005 ▶); cell refinement: *SAINT* (Bruker, 2005 ▶); data reduction: *SAINT*; program(s) used to solve structure: *SHELXS97* (Sheldrick, 2008 ▶); program(s) used to refine structure: *SHELXL97* (Sheldrick, 2008 ▶); molecular graphics: *SHELXTL* (Sheldrick, 2008 ▶); software used to prepare material for publication: *SHELXTL*.

## Supplementary Material

Crystal structure: contains datablock(s) I, global. DOI: 10.1107/S1600536812043875/gg2103sup1.cif

Structure factors: contains datablock(s) I. DOI: 10.1107/S1600536812043875/gg2103Isup2.hkl

Supplementary material file. DOI: 10.1107/S1600536812043875/gg2103Isup3.cml

Additional supplementary materials:  crystallographic information; 3D view; checkCIF report

## Figures and Tables

**Table 1:** Hydrogen-bond geometry (Å, °) *Cg*1 and *Cg*2 are the centroids of the C4–C9 and C20–C25 rings, respectively.

*D*—H⋯*A*	*D*—H	H⋯*A*	*D*⋯*A*	*D*—H⋯*A*
C5—H5⋯N2^i^	0.93	2.62	3.517 (4)	161
C16—H16⋯O1^ii^	0.93	2.58	3.427 (4)	152
C21—H21⋯N4^iii^	0.93	2.62	3.513 (4)	161
C32—H32⋯O2^ii^	0.93	2.57	3.410 (4)	150
C1—H1*C*⋯*Cg*1^iv^	0.96	2.61	3.487 (4)	151
C17—H17*A*⋯*Cg*2^iv^	0.96	2.61	3.491 (4)	153

Symmetry codes: (i) 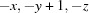; (ii) 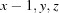; (iii) 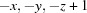; (iv) 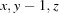.

**Fig. 1.**
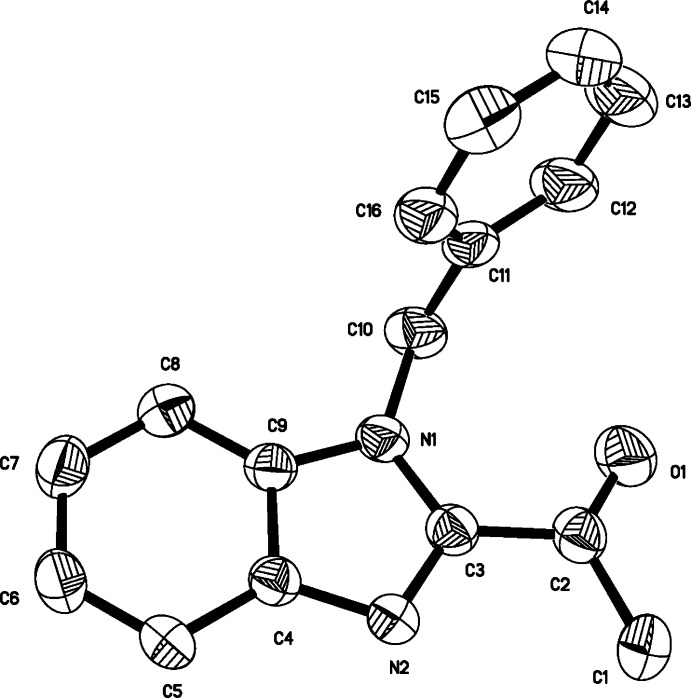
The molecular structure of (I), showing displacement ellipsoids drawn at the 50% probability level.

## supplementary crystallographic information

### Comment

Synthesis of nitrogen-containing heterocyclic compounds has been a subject of great interest due to the wide applications in the agrochemical and pharmaceutical fields (Ge *et al.*; 2009, 2011). Some benzoimidazole derivatives which belong to this category exhibit interesting biological properties, such as anti-bacterial, anti-inflammatory, anti-fungal and anti-tumor. The title benzoimidazole(I) (Fig. 1) was synthesized in order to study its biological properties. (I) was screened for anticancer activities and found to be inactive.

We report here the crystal structure of the title compound. In the molecular structure, the 90 degree angle on alpha shows the benzene ring and the imidazole are in the same plane and the two benzene ring makes dihedral angle of 85.92 (8)°. Moreover, there exist inermolecular weak C—H···O and C—H···N hydrogen bonding, also the intermolecular face-to-face C—H···π stacking interaction.

### Experimental

A mixture of 1-(1*H*-benzo[*d*]imidazol-2-yl)ethanone(0.02 mol), (chloromethyl)benzene (0.024 mol) and potassium carbonate (0.024 mol) in acetonitrile (100 ml) was heated to reflux for 5 h. The solvent was removed under reduced pressure and the product was isolated by column chromatography on silica gel (yield 85%). Crystals of (I) suitable for X-ray diffraction were obtained by allowing a refluxed solution of the product in ethyl acetate (0.10 *M*) to cool slowly to room temperature (without temperature control) and allowing the solvent to evaporate for 12 h.

### Refinement

All H atoms were placed in geometrically calculated positions and refined using a riding model with C—H = 0.97 Å (for CH_2_ groups) and 0.93 Å (for aromatic protons), their isotropic displacement parameters were set to 1.2 times the equivalent displacement parameter of their parent atoms.

### Crystal data

**Table:**

C_16_H_14_N_2_O	*Z* = 4
*M**_r_* = 250.29	*F*(000) = 528
Triclinic, *P*1	*D*_x_ = 1.281 Mg m^−^^3^
Hall symbol: -P 1	Mo *K*α radiation, λ = 0.71073 Å
*a* = 6.1307 (10) Å	Cell parameters from 3788 reflections
*b* = 6.5226 (12) Å	θ = 2.9–28.3°
*c* = 34.739 (6) Å	µ = 0.08 mm^−^^1^
α = 90.021 (3)°	*T* = 293 K
β = 92.749 (3)°	Block, colorless
γ = 110.674 (3)°	0.28 × 0.24 × 0.19 mm
*V* = 1298.0 (4) Å^3^	

### Data collection

**Table:**

Brucker APEXII CCD area-detector diffractometer	4523 independent reflections
Radiation source: fine-focus sealed tube	3775 reflections with *I* > 2σ(*I*)
Graphite monochromator	*R*_int_ = 0.061
φ and ω scans	θ_max_ = 25.1°, θ_min_ = 2.4°
Absorption correction: multi-scan (*SADABS*; Bruker, 2005)	*h* = −7→5
*T*_min_ = 0.978, *T*_max_ = 0.985	*k* = −6→7
6666 measured reflections	*l* = −41→40

### Refinement

**Table:**

Refinement on *F*^2^	Primary atom site location: structure-invariant direct methods
Least-squares matrix: full	Secondary atom site location: difference Fourier map
*R*[*F*^2^ > 2σ(*F*^2^)] = 0.068	Hydrogen site location: inferred from neighbouring sites
*wR*(*F*^2^) = 0.189	H-atom parameters constrained
*S* = 1.05	*w* = 1/[σ^2^(*F*_o_^2^) + (0.080*P*)^2^ + 1.3353*P*] where *P* = (*F*_o_^2^ + 2*F*_c_^2^)/3
4523 reflections	(Δ/σ)_max_ = 0.009
345 parameters	Δρ_max_ = 0.23 e Å^−^^3^
0 restraints	Δρ_min_ = −0.29 e Å^−^^3^

### Special details

**Table:**

Geometry. All e.s.d.'s (except the e.s.d. in the dihedral angle between two l.s. planes) are estimated using the full covariance matrix. The cell e.s.d.'s are taken into account individually in the estimation of e.s.d.'s in distances, angles and torsion angles; correlations between e.s.d.'s in cell parameters are only used when they are defined by crystal symmetry. An approximate (isotropic) treatment of cell e.s.d.'s is used for estimating e.s.d.'s involving l.s. planes.
Refinement. Refinement of *F*^2^ against ALL reflections. The weighted *R*-factor *wR* and goodness of fit *S* are based on *F*^2^, conventional *R*-factors *R* are based on *F*, with *F* set to zero for negative *F*^2^. The threshold expression of *F*^2^ > σ(*F*^2^) is used only for calculating *R*-factors(gt) *etc*. and is not relevant to the choice of reflections for refinement. *R*-factors based on *F*^2^ are statistically about twice as large as those based on *F*, and *R*- factors based on ALL data will be even larger.

### Fractional atomic coordinates and isotropic or equivalent isotropic displacement parameters (Å^2^)

**Table:**

	*x*	*y*	*z*	*U*_iso_*/*U*_eq_	
N2	0.2590 (4)	0.5385 (4)	0.04941 (6)	0.0375 (5)	
N1	0.3603 (4)	0.6559 (4)	0.11075 (6)	0.0387 (5)	
C9	0.2089 (5)	0.7550 (4)	0.09724 (8)	0.0360 (6)	
C4	0.1469 (5)	0.6810 (4)	0.05892 (8)	0.0349 (6)	
O1	0.6433 (4)	0.3837 (4)	0.11196 (7)	0.0618 (7)	
C3	0.3821 (5)	0.5266 (5)	0.08078 (8)	0.0374 (6)	
C11	0.3396 (5)	0.4803 (5)	0.17452 (8)	0.0431 (7)	
C8	0.1216 (6)	0.9027 (5)	0.11414 (9)	0.0457 (7)	
H8	0.1639	0.9531	0.1394	0.055*	
C10	0.4598 (6)	0.6760 (5)	0.15034 (8)	0.0478 (7)	
H10A	0.4493	0.8061	0.1624	0.057*	
H10B	0.6239	0.6952	0.1497	0.057*	
C5	−0.0061 (5)	0.7522 (5)	0.03642 (8)	0.0415 (7)	
H5	−0.0477	0.7043	0.0110	0.050*	
C2	0.5329 (5)	0.3922 (5)	0.08265 (9)	0.0426 (7)	
C1	0.5351 (6)	0.2674 (5)	0.04673 (10)	0.0533 (8)	
H1A	0.6700	0.2249	0.0478	0.080*	
H1B	0.5401	0.3581	0.0248	0.080*	
H1C	0.3965	0.1388	0.0445	0.080*	
C6	−0.0931 (6)	0.8958 (5)	0.05327 (9)	0.0481 (7)	
H6	−0.1964	0.9448	0.0389	0.058*	
C7	−0.0307 (6)	0.9703 (5)	0.09136 (10)	0.0497 (8)	
H7	−0.0933	1.0679	0.1017	0.060*	
C16	0.0981 (6)	0.3821 (5)	0.17316 (9)	0.0487 (7)	
H16	0.0072	0.4360	0.1568	0.058*	
C12	0.4703 (7)	0.3977 (7)	0.19917 (9)	0.0612 (9)	
H12	0.6322	0.4621	0.2005	0.073*	
C13	0.3631 (8)	0.2209 (8)	0.22184 (11)	0.0769 (12)	
H13	0.4536	0.1660	0.2381	0.092*	
C14	0.1242 (8)	0.1247 (7)	0.22070 (10)	0.0694 (11)	
H14	0.0527	0.0070	0.2364	0.083*	
C15	−0.0081 (7)	0.2042 (6)	0.19608 (10)	0.0605 (9)	
H15	−0.1699	0.1382	0.1948	0.073*	
N3	0.2918 (4)	0.1314 (4)	0.38915 (6)	0.0382 (5)	
N4	0.2276 (4)	0.0279 (4)	0.45054 (6)	0.0373 (5)	
C25	0.1488 (5)	0.2343 (4)	0.40291 (8)	0.0365 (6)	
C20	0.1103 (5)	0.1685 (4)	0.44105 (7)	0.0343 (6)	
O2	0.5754 (4)	−0.1398 (4)	0.38807 (7)	0.0604 (6)	
C19	0.3329 (5)	0.0094 (4)	0.41922 (8)	0.0367 (6)	
C27	0.2311 (5)	−0.0590 (5)	0.32567 (8)	0.0420 (7)	
C21	−0.0289 (5)	0.2441 (5)	0.46367 (8)	0.0420 (7)	
H21	−0.0546	0.2018	0.4891	0.050*	
C26	0.3662 (5)	0.1418 (5)	0.34957 (8)	0.0463 (7)	
H26A	0.5306	0.1609	0.3502	0.056*	
H26B	0.3482	0.2689	0.3374	0.056*	
C18	0.4815 (5)	−0.1260 (5)	0.41743 (9)	0.0429 (7)	
C24	0.0503 (5)	0.3784 (5)	0.38595 (8)	0.0447 (7)	
H24	0.0760	0.4228	0.3606	0.054*	
C17	0.5069 (6)	−0.2427 (5)	0.45347 (10)	0.0511 (8)	
H17A	0.3755	−0.3767	0.4548	0.077*	
H17B	0.5136	−0.1511	0.4755	0.077*	
H17C	0.6479	−0.2751	0.4533	0.077*	
C28	0.3418 (7)	−0.1542 (6)	0.30162 (9)	0.0580 (9)	
H28	0.5033	−0.0937	0.3005	0.070*	
C32	−0.0115 (6)	−0.1540 (6)	0.32685 (9)	0.0495 (8)	
H32	−0.0909	−0.0935	0.3429	0.059*	
C23	−0.0862 (6)	0.4507 (5)	0.40859 (9)	0.0479 (7)	
H23	−0.1541	0.5471	0.3984	0.058*	
C22	−0.1260 (6)	0.3834 (5)	0.44664 (9)	0.0483 (7)	
H22	−0.2214	0.4349	0.4609	0.058*	
C31	−0.1339 (7)	−0.3367 (6)	0.30447 (11)	0.0619 (9)	
H31	−0.2954	−0.3985	0.3055	0.074*	
C29	0.2199 (8)	−0.3370 (7)	0.27911 (10)	0.0714 (11)	
H29	0.2983	−0.3982	0.2630	0.086*	
C30	−0.0193 (8)	−0.4277 (7)	0.28074 (11)	0.0697 (11)	
H30	−0.1030	−0.5510	0.2657	0.084*	

### Atomic displacement parameters (Å^2^)

**Table:**

	*U*^11^	*U*^22^	*U*^33^	*U*^12^	*U*^13^	*U*^23^
N2	0.0412 (13)	0.0429 (12)	0.0320 (12)	0.0194 (10)	0.0003 (10)	0.0024 (9)
N1	0.0393 (13)	0.0487 (13)	0.0301 (12)	0.0185 (11)	−0.0017 (10)	0.0009 (10)
C9	0.0356 (14)	0.0407 (14)	0.0315 (14)	0.0131 (12)	0.0028 (11)	0.0027 (11)
C4	0.0361 (14)	0.0363 (13)	0.0326 (13)	0.0132 (11)	0.0029 (11)	0.0035 (11)
O1	0.0563 (14)	0.0921 (18)	0.0516 (14)	0.0450 (14)	−0.0029 (11)	0.0094 (12)
C3	0.0351 (14)	0.0423 (14)	0.0358 (14)	0.0149 (12)	0.0023 (11)	0.0040 (11)
C11	0.0473 (17)	0.0631 (18)	0.0257 (13)	0.0283 (15)	−0.0018 (12)	−0.0035 (12)
C8	0.0525 (18)	0.0479 (16)	0.0392 (16)	0.0208 (14)	0.0034 (13)	−0.0027 (13)
C10	0.0443 (17)	0.0619 (19)	0.0340 (15)	0.0162 (14)	−0.0093 (13)	−0.0031 (13)
C5	0.0471 (17)	0.0460 (16)	0.0345 (14)	0.0211 (13)	−0.0027 (12)	0.0053 (12)
C2	0.0354 (15)	0.0516 (17)	0.0443 (17)	0.0196 (13)	0.0040 (13)	0.0078 (13)
C1	0.059 (2)	0.0536 (18)	0.058 (2)	0.0321 (16)	0.0067 (16)	0.0002 (15)
C6	0.0492 (18)	0.0515 (17)	0.0515 (18)	0.0278 (15)	0.0000 (14)	0.0079 (14)
C7	0.0538 (19)	0.0478 (17)	0.0553 (19)	0.0271 (15)	0.0085 (15)	0.0009 (14)
C16	0.0509 (18)	0.0628 (19)	0.0368 (16)	0.0259 (16)	−0.0005 (13)	0.0005 (14)
C12	0.058 (2)	0.096 (3)	0.0390 (17)	0.039 (2)	0.0002 (15)	0.0114 (17)
C13	0.085 (3)	0.111 (3)	0.054 (2)	0.057 (3)	0.007 (2)	0.030 (2)
C14	0.095 (3)	0.076 (2)	0.046 (2)	0.040 (2)	0.0195 (19)	0.0160 (17)
C15	0.058 (2)	0.067 (2)	0.057 (2)	0.0204 (17)	0.0105 (16)	−0.0019 (17)
N3	0.0403 (13)	0.0476 (13)	0.0287 (11)	0.0178 (11)	0.0051 (9)	0.0045 (9)
N4	0.0401 (13)	0.0428 (13)	0.0325 (12)	0.0188 (10)	0.0039 (10)	0.0037 (9)
C25	0.0363 (14)	0.0391 (14)	0.0344 (14)	0.0136 (12)	0.0019 (11)	0.0017 (11)
C20	0.0379 (14)	0.0357 (13)	0.0296 (13)	0.0130 (11)	0.0022 (11)	0.0015 (10)
O2	0.0567 (14)	0.0883 (17)	0.0514 (14)	0.0436 (13)	0.0108 (11)	−0.0015 (12)
C19	0.0355 (14)	0.0406 (14)	0.0349 (14)	0.0149 (12)	0.0010 (11)	0.0024 (11)
C27	0.0492 (17)	0.0603 (18)	0.0256 (13)	0.0303 (14)	0.0049 (12)	0.0091 (12)
C21	0.0478 (17)	0.0451 (16)	0.0370 (15)	0.0205 (13)	0.0069 (12)	0.0013 (12)
C26	0.0446 (17)	0.0598 (18)	0.0353 (15)	0.0181 (14)	0.0120 (13)	0.0125 (13)
C18	0.0364 (15)	0.0513 (17)	0.0436 (17)	0.0190 (13)	−0.0001 (13)	−0.0014 (13)
C24	0.0523 (18)	0.0495 (16)	0.0349 (15)	0.0219 (14)	−0.0015 (13)	0.0072 (12)
C17	0.0552 (19)	0.0553 (18)	0.0538 (19)	0.0334 (16)	0.0008 (15)	0.0070 (14)
C28	0.062 (2)	0.092 (3)	0.0353 (16)	0.046 (2)	0.0044 (15)	0.0034 (16)
C32	0.0486 (18)	0.065 (2)	0.0413 (16)	0.0274 (16)	0.0057 (13)	0.0009 (14)
C23	0.0519 (18)	0.0477 (17)	0.0516 (18)	0.0275 (14)	−0.0025 (14)	0.0054 (14)
C22	0.0525 (18)	0.0511 (17)	0.0502 (18)	0.0288 (15)	0.0056 (14)	−0.0019 (14)
C31	0.058 (2)	0.071 (2)	0.057 (2)	0.0253 (18)	−0.0066 (17)	0.0025 (17)
C29	0.095 (3)	0.099 (3)	0.0431 (19)	0.063 (3)	−0.0003 (19)	−0.0115 (19)
C30	0.089 (3)	0.079 (3)	0.050 (2)	0.043 (2)	−0.0192 (19)	−0.0122 (18)

### Geometric parameters (Å, º)

**Table:**

N2—C3	1.313 (4)	N3—C25	1.379 (4)
N2—C4	1.385 (4)	N3—C19	1.380 (4)
N1—C9	1.372 (4)	N3—C26	1.464 (4)
N1—C3	1.380 (4)	N4—C19	1.318 (4)
N1—C10	1.464 (4)	N4—C20	1.382 (4)
C9—C8	1.398 (4)	C25—C24	1.401 (4)
C9—C4	1.404 (4)	C25—C20	1.398 (4)
C4—C5	1.394 (4)	C20—C21	1.397 (4)
O1—C2	1.208 (4)	O2—C18	1.213 (4)
C3—C2	1.481 (4)	C19—C18	1.480 (4)
C11—C12	1.380 (4)	C27—C28	1.377 (4)
C11—C16	1.389 (5)	C27—C32	1.397 (4)
C11—C10	1.508 (4)	C27—C26	1.503 (4)
C8—C7	1.383 (4)	C21—C22	1.371 (4)
C8—H8	0.9300	C21—H21	0.9300
C10—H10A	0.9700	C26—H26A	0.9700
C10—H10B	0.9700	C26—H26B	0.9700
C5—C6	1.373 (4)	C18—C17	1.495 (4)
C5—H5	0.9300	C24—C23	1.372 (4)
C2—C1	1.493 (4)	C24—H24	0.9300
C1—H1A	0.9600	C17—H17A	0.9600
C1—H1B	0.9600	C17—H17B	0.9600
C1—H1C	0.9600	C17—H17C	0.9600
C6—C7	1.398 (5)	C28—C29	1.380 (6)
C6—H6	0.9300	C28—H28	0.9300
C7—H7	0.9300	C32—C31	1.378 (5)
C16—C15	1.386 (5)	C32—H32	0.9300
C16—H16	0.9300	C23—C22	1.398 (5)
C12—C13	1.378 (6)	C23—H23	0.9300
C12—H12	0.9300	C22—H22	0.9300
C13—C14	1.373 (6)	C31—C30	1.370 (6)
C13—H13	0.9300	C31—H31	0.9300
C14—C15	1.375 (5)	C29—C30	1.378 (6)
C14—H14	0.9300	C29—H29	0.9300
C15—H15	0.9300	C30—H30	0.9300
			
C3—N2—C4	105.1 (2)	C25—N3—C19	106.0 (2)
C9—N1—C3	106.3 (2)	C25—N3—C26	125.3 (2)
C9—N1—C10	124.9 (2)	C19—N3—C26	128.6 (2)
C3—N1—C10	128.7 (2)	C19—N4—C20	105.3 (2)
N1—C9—C8	132.5 (3)	N3—C25—C24	132.4 (3)
N1—C9—C4	105.9 (2)	N3—C25—C20	106.1 (2)
C8—C9—C4	121.6 (3)	C24—C25—C20	121.5 (3)
N2—C4—C5	129.5 (2)	N4—C20—C21	129.3 (2)
N2—C4—C9	109.7 (2)	N4—C20—C25	109.7 (2)
C5—C4—C9	120.8 (3)	C21—C20—C25	121.0 (3)
N2—C3—N1	113.0 (2)	N4—C19—N3	112.8 (2)
N2—C3—C2	122.5 (3)	N4—C19—C18	122.6 (2)
N1—C3—C2	124.5 (2)	N3—C19—C18	124.5 (2)
C12—C11—C16	118.7 (3)	C28—C27—C32	117.8 (3)
C12—C11—C10	119.9 (3)	C28—C27—C26	121.2 (3)
C16—C11—C10	121.5 (3)	C32—C27—C26	121.0 (3)
C7—C8—C9	116.7 (3)	C22—C21—C20	116.9 (3)
C7—C8—H8	121.7	C22—C21—H21	121.5
C9—C8—H8	121.7	C20—C21—H21	121.5
N1—C10—C11	113.2 (2)	N3—C26—C27	113.3 (2)
N1—C10—H10A	108.9	N3—C26—H26A	108.9
C11—C10—H10A	108.9	C27—C26—H26A	108.9
N1—C10—H10B	108.9	N3—C26—H26B	108.9
C11—C10—H10B	108.9	C27—C26—H26B	108.9
H10A—C10—H10B	107.7	H26A—C26—H26B	107.7
C6—C5—C4	117.4 (3)	O2—C18—C19	120.9 (3)
C6—C5—H5	121.3	O2—C18—C17	123.0 (3)
C4—C5—H5	121.3	C19—C18—C17	116.2 (3)
O1—C2—C3	121.0 (3)	C23—C24—C25	116.7 (3)
O1—C2—C1	122.9 (3)	C23—C24—H24	121.7
C3—C2—C1	116.0 (3)	C25—C24—H24	121.7
C2—C1—H1A	109.5	C18—C17—H17A	109.5
C2—C1—H1B	109.5	C18—C17—H17B	109.5
H1A—C1—H1B	109.5	H17A—C17—H17B	109.5
C2—C1—H1C	109.5	C18—C17—H17C	109.5
H1A—C1—H1C	109.5	H17A—C17—H17C	109.5
H1B—C1—H1C	109.5	H17B—C17—H17C	109.5
C5—C6—C7	121.9 (3)	C27—C28—C29	121.8 (4)
C5—C6—H6	119.1	C27—C28—H28	119.1
C7—C6—H6	119.1	C29—C28—H28	119.1
C8—C7—C6	121.7 (3)	C31—C32—C27	120.5 (3)
C8—C7—H7	119.2	C31—C32—H32	119.7
C6—C7—H7	119.2	C27—C32—H32	119.7
C15—C16—C11	120.3 (3)	C24—C23—C22	121.7 (3)
C15—C16—H16	119.9	C24—C23—H23	119.1
C11—C16—H16	119.9	C22—C23—H23	119.1
C13—C12—C11	120.6 (4)	C21—C22—C23	122.1 (3)
C13—C12—H12	119.7	C21—C22—H22	118.9
C11—C12—H12	119.7	C23—C22—H22	118.9
C14—C13—C12	120.8 (4)	C30—C31—C32	120.4 (4)
C14—C13—H13	119.6	C30—C31—H31	119.8
C12—C13—H13	119.6	C32—C31—H31	119.8
C15—C14—C13	119.3 (4)	C28—C29—C30	119.3 (3)
C15—C14—H14	120.4	C28—C29—H29	120.3
C13—C14—H14	120.4	C30—C29—H29	120.3
C14—C15—C16	120.4 (4)	C31—C30—C29	120.1 (4)
C14—C15—H15	119.8	C31—C30—H30	119.9
C16—C15—H15	119.8	C29—C30—H30	119.9

### Hydrogen-bond geometry (Å, º)

Cg1 and Cg2 are the centroids of the C4–C9 and C20–C25 rings, respectively.

**Table:**

*D*—H···*A*	*D*—H	H···*A*	*D*···*A*	*D*—H···*A*
C5—H5···N2^i^	0.93	2.62	3.517 (4)	161
C10—H10*B*···O1	0.97	2.46	2.887 (4)	106
C16—H16···O1^ii^	0.93	2.58	3.427 (4)	152
C21—H21···N4^iii^	0.93	2.62	3.513 (4)	161
C26—H26*A*···O2	0.97	2.45	2.882 (4)	107
C32—H32···O2^ii^	0.93	2.57	3.410 (4)	150
C1—H1*C*···*Cg*1^iv^	0.96	2.61	3.487 (4)	151
C17—H17*A*···*Cg*2^iv^	0.96	2.61	3.491 (4)	153

Symmetry codes: (i) −*x*, −*y*+1, −*z*; (ii) *x*−1, *y*, *z*; (iii) −*x*, −*y*, −*z*+1; (iv) *x*, *y*−1, *z*.
